# Untargeted Metabolomics to Evaluate the Stability of Extra-Virgin Olive Oil with Added *Lycium barbarum* Carotenoids during Storage

**DOI:** 10.3390/foods8060179

**Published:** 2019-05-28

**Authors:** Domenico Montesano, Gabriele Rocchetti, Lina Cossignani, Biancamaria Senizza, Luna Pollini, Luigi Lucini, Francesca Blasi

**Affiliations:** 1 Department of Pharmaceutical Sciences, Section of Food Science and Nutrition, University of Perugia, via San Costanzo, 06126 Perugia, Italy; domenico.montesano@unipg.it (D.M.); luna.pollini@studenti.unipg.it (L.P.); francesca.blasi@unipg.it (F.B.); 2 Department for Sustainable Food Process, Università Cattolica del Sacro Cuore, Via Emilia Parmense 84, 29122 Piacenza, Italy; gabriele.rocchetti@unicatt.it (G.R.); biancam.senizza@virgilio.it (B.S.); luigi.lucini@unicatt.it (L.L.)

**Keywords:** carotenoids, Goji berries, metabolomics, long-term storage, olive oil, oxidative stability

## Abstract

A carotenoid-rich extract from *Lycium barbarum* L. was added to extra virgin olive oil (EVOO), obtaining a carotenoid-enriched oil (EVOOCar). The oxidative stability of EVOO and EVOOCar was evaluated during long-term storage of 28 weeks at room temperature, by measuring some classical parameters (acidity and peroxide values, spectrophotometric coefficients, fatty acid composition) and the content of minor compounds (i.e., α-tocopherol and lutein). At the end of the storage, higher content (*p* < 0.01) of α-tocopherol in EVOOCar in respect to EVOO were observed. Zeaxanthin dipalmitate, the most abundant carotenoid compound of Goji berries, decreased slightly (*p* < 0.05) in EVOOCar until the end of the storage. In regard to polyphenols, an ultra-high-performance liquid chromatography coupled to quadrupole time-of-flight mass spectrometry (UHPLC/QTOF-MS) using untargeted metabolomics was carried out. This latter approach discriminated the two oil samples during long-term storage, allowing to identify also the phenolic classes most exposed to significant variations during storage (i.e., mainly lignans and flavones). Besides, the addition of Goji carotenoids preserved the stability of tyrosol equivalents in EVOOCar during long-term storage. These results highlighted that the enrichment of EVOO with a carotenoid-rich extract can improve the shelf-life and nutritional value of added-oil, protecting EVOO natural antioxidants during long-term storage.

## 1. Introduction

Food lipids are prone to undergo oxidation phenomena during storage and technological and household treatments. In order to prevent/avoid their oxidative degradation, the most popular method is the addition of antioxidants. Two kinds of antioxidants can be used: synthetic and natural [1]. They may be added to prevent or minimize oil oxidative deterioration, even if, the use of synthetic antioxidant additives in foods (i.e., butylated hydroxytoluene (BHT), butylated hydroxyanisole (BHA), and *tert*-butyl hydroquinone) is restricted and regulated by the European Food Safety Authority [2], the U.S. Food and Drug Administration [3], and the Food and Agriculture Organization of the United Nations [4]. Moreover, the addition of synthetic antioxidants might cause undesirable consequences for the olive oil quality [5] and might cause toxicity including carcinogenesis [6]. It was reported that the allowed doses of BHT and BHA do not provide enough oxidative stability for oils [7].

As a result, nowadays, the research of natural antioxidants, for improving oil oxidative stability and for replacing synthetic antioxidants, has attracted more and more attention from researchers [1,8]. When compared to synthetic antioxidants, natural antioxidants have many advantages [9]. They are considered to be safe and readily acceptable by consumers. In addition, these antioxidants not only stabilize edible oils, but also improve the nutraceutical value in order to obtain a value-added product [10].

In this context, many recent papers have reported that spices, herbs (used in the form of ground materials or extracts), essential oils, and vegetable extracts [8,10,11,12], if added to edible oils, are able to hinder the oxidative degradation. Extra virgin olive oil (EVOO), the most important edible oil of the Mediterranean diet, presents better stability against oxidation and heating processes in comparison to other edible oils [13]. Its nutritional characteristics and health benefits are primarily related to its native antioxidant fraction and to the high content of monounsaturated fatty acids (MUFA), which confer to EVOO higher resistance against stress oxidative and heat processes [14].

Nevertheless, various papers [11,15,16,17] report on the enrichment of EVOO in order to improve its nutritional value and to ameliorate its chemical stability. For example, EVOO samples, added with oregano essential oil, showed improved stability in different conditions (darkness, light exposure, and 60 °C), with respect to non-added EVOO [15]. The addition of the essential oil of *Zataria multiflora* (Shirazi thyme) reduced the EVOO oxidation to the same extent as BHT, and more effectively than α-tocopherol [11]. Recently, some researchers observed that EVOO can be preserved against oxidation using sesame seed oil during storage [16].

In addition to EVOO, other oils have been treated. The oxidative stability of soybean, rice bran and cottonseed oils added with rosemary extract were tested during 24 days of storage at 62 °C [18]. Recently, the oxidative stability of sunflower oil flavored by essential oil from *Coriandrum sativum* L. during accelerated storage has been studied [8]. The effectiveness of the addition of some natural antioxidants (citric acid, ascorbyl palmitate, α-tocopherol, δ-tocopherol, and ascorbic acid) in sunflower oil, stored for different times at different temperatures was studied [19].

In a previous work [17], it has been reported that the addition of a carotenoid-rich extract from *Lycium barbarum* L. can help to improve the oxidative stability of EVOO during a frying process. In recent years, the interest in *L. barbarum* fruit increased, due to its high amount of bioactive compounds including polyphenols [20], fatty acids [21,22], phytosterols [23], minerals, and carotenoids [24], but also simple sugars [25]. The main object of this work was to study the effect of EVOO enrichment with a carotenoid-rich extract from *L. barbarum* in terms of chemical-physical change and heat stability during long-term storage. Besides the known chemical parameters typically considered in oil thermal stability assessments, our work aimed to investigate the changes of the main phenolic classes by applying a metabolomic approach based on untargeted ultra-high-performance liquid chromatography coupled to quadrupole time-of-flight mass spectrometry (UHPLC/QTOF-MS).

## 2. Materials and Methods

### 2.1. Raw Materials and Reagents

Italian Goji berry samples, collected in 2016, were provided by the Favella Group of Rizzo Nicola (Corigliano Calabro, Cosenza, Italy). The EVOO sample was purchased in November 2016 from an Umbrian farm (Perugia, Central Italy). BHT (≥99.0%) and α-tocopherol (≥96.0%) analytical standards were purchased from Sigma-Aldrich (St. Louis, MO, USA). Lutein (≥95.0%) and all-*trans*-zeaxanthin (≥98.0%) analytical standards were from Extrasynthese (Lyon, France). Supelco™ 37 component fatty acid methyl esters (FAME) mix (catalog n. 47885-U), containing the methyl esters of 37 fatty acids (the FA contents ranged between 2% and 4%, while the palmitic acid methyl ester was 6%), was bought from Supelco (Bellefonte, PA, USA). Methyl *tert*-butyl ether (MTBE), water (proteomic grade), methanol and acetonitrile (liquid chromatography-mass spectrometry grade) were from VWR (Milan, Italy). The other solvents were purchased from Carlo Erba Reagents (Milan, Italy).

### 2.2. Extraction of Carotenoids from Goji Berries

A carotenoid-rich extract from Goji was obtained using a procedure developed in a previous work [17]. In brief, dried *L. barbarum* berries were treated with distilled water and then with absolute methanol in a blender, then centrifuged and filtered. The recovered residue was added with hexane/acetone (3:2, *v*:*v*) and sonicated, then the solution was filtered under vacuum. This procedure was repeated until the residue became colorless. After that, the extracted fractions were pooled and dried over anhydrous sodium sulfate. The final residue was weighed, dissolved in MTBE, and immediately analyzed by high-performance liquid chromatography-mass spectrometry chromatography-diode array detector-mass spectrometry (HPLC-DAD-MS), as described in Section 2.7.

### 2.3. Preparation of EVOO Sample Added with Goji Carotenoids

Carotenoids extracted from Goji were added to EVOO at a concentration of 1.5 mg/100 g oil (EVOOCar), according to a previous paper [26]. To improve the extract dispersion in oil, the EVOOCar sample was sonicated for 2 min and then shaded for 10 min. Finally, the sample appeared homogeneous and without particulate matter. EVOO and EVOOCar were immediately analyzed and represented the sample at time zero.

### 2.4. Storage Conditions

Aliquots of EVOO and EVOOCar samples were stored in 30-mL amber glass bottles, in the dark at 20 °C for 28 weeks. Three 30 mL aliquots of EVOO and three 30 mL aliquots of EVOOCar have been prepared for each storage time, including the samples at time zero. The samples were analyzed every 4 weeks up to 28 weeks; the samples were also stored at −20 °C to complete the analyses. Oil stability was evaluated by measuring acidity value (AV), peroxide value (PV), specific absorptivity at 232 and 270 nm, fatty acid (FA) composition, and carotenoid and α-tocopherol contents. Furthermore, comprehensive phenolic profiling, based on UHPLC/QTOF-MS, was used.

### 2.5. Determination of Acidity Value, Peroxide Value, and Specific Extinction Values

Classical parameters (AV, PV, and specific extinction values) were determined following the AOAC method [27], as previously reported [17] with slight modifications.

### 2.6. High Resolution Gas Chromatography Analysis of Fatty Acid Methyl Esters

The fatty acid methyl esters (FAME) of triacylglycerols (TAG) were prepared by transesterification as reported in a previous work [28]. The analysis of FAME was carried out using a Dani GC1000 gas chromatograph (Norwalk, CT, USA) equipped with a split/splitless injector port and a Flame Ionization Detector. The fused silica WCOT capillary column CP-Select CB for FAME (50 m × 0.25 mm i.d., 0.25 μm f.t., Varian, Superchrom, Milan, Italy) was used. The chromatograms were acquired and processed using Clarity integration software (DataApex Ltd., Prague, Czech Republic). The chromatographic conditions were the following: The injector and detector temperature was 250 °C; the oven temperature was 180 °C, held for 6 min then increased to 250 °C at 3 °C/min; the final temperature was held for 10 min. The carrier gas (He) flow rate was 1.0 mL/min.

### 2.7. HPLC-DAD-MS Analysis of Carotenoids and α-Tocopherol

The quali-quantitative determination of carotenoids was performed by reversed phase chromatography (RP-HPLC-MS) method, as reported in a previous work [29]. In brief, a Spectra System HPLC system (Thermo Separation, San Jose, CA, USA) was employed, equipped with a quaternary pump module (P4000), an online degasser, and a Spectra System UV 6000lp DAD (Thermo Separation) coupled with an electrospray ionization (ESI) MS detector in positive ion mode (Finnigan LC Aqua, Finnigan, Manchester, UK).

Carotenoids, lutein, zeaxanthin dipalmitate, and α-tocopherol were eluted using a C30 column (250 × 4.6 mm, 5 μm; Nomura Co., Kyoto, Japan) coupled to a C30 guard column (20 × 4.6 mm) using mobile phases consisting of methanol and MTBE. The conditions for the analysis and the validation of the method were reported in a previous work [17]. Peaks were identified by comparing the retention times, UV-Vis spectral data, and MS data with those of the corresponding standards and literature [26,30]. Detection was performed online using the DAD in the range from 200 to 700 nm (carotenoids analysis was performed at 450 nm, α-tocopherol at 292 nm). MS detection was carried out with a scan range of *m*/*z* 100–1200. Chromatograms and spectra were elaborated with an Xcalibur software 2.2 data system (Thermo Fisher Scientific, Pittsburgh, PA, USA). All compounds were quantified using the calibration curve built with the respective standard, except zeaxanthin dipalmitate that was dosed using all-*trans*-zeaxanthin calibration curve.

### 2.8. UHPLC/QTOF Screening of Phenolic Compounds 

Polyphenols were extracted in triplicate from each EVOO sample according to a previously published method [31]. Briefly, an aliquot (3 g) of oil was accurately weighted into conical centrifuge tubes and 3 mL of 80% methanol solution (*v/v*) (LCMS grade, VWR, Milan Italy) was added. The mixtures were vortexed vigorously and then centrifuged at 6000× *g* for 10 min at 4 °C. The methanol fractions were collected, whilst the residues were discarded. The resulting supernatants were filtered through 0.22 μm cellulose syringe filters and stored in amber vials at −20 °C until the following analysis.

Afterward, the phenolic compounds in EVOO samples collected after 0, 20, and 28 weeks of storage were screened through UHPLC/QTOF-MS, using a 1290 liquid chromatograph coupled with a G6550 mass spectrometer detector, via a Dual JetStream Electrospray Ionization System (all from Agilent Technologies, Santa Clara, CA, USA). UHPLC/QTOF-MS analytical conditions for the analysis of phenolic compounds in EVOO samples were optimized in previous experiments [20,32]. Briefly, the mass spectrometer was set up in positive scan mode, to acquire the range of 50–1200 *m/z*. Reverse phase chromatography was carried out using a Knauer Blue Orchid C18 column (100 × 2 mm i.d., 1.8 μm) and a mixture of water and acetonitrile as mobile phases. Formic acid 0.1% (*v/v*) was then added to both phases. The elution gradient started from 5% acetonitrile and was increased to 90% acetonitrile within 34 min, the flow rate was 220 μL/min, and the injection volume was 6 μL.

The raw data were then processed using the Agilent Profinder B.07 software, according to the ‘find-by-formula’ algorithm. Monoisotopic accurate mass was used together with the whole isotope pattern (isotopic spacing and isotopic ratio) to achieve a higher confidence for identification. The database exported from Phenol-Explorer 3.6 was used as a reference for identification, adopting a 5-ppm tolerance for mass accuracy. Recursive identification and filter-by-frequency were applied as post-acquisition processing: features that were not present in 100% replications within at least one treatment were discarded. This processed dataset was finally used for statistics and chemometrics purposes. Furthermore, polyphenols annotated were ascribed into classes and sub-classes and then cumulative intensities were calculated. Methanolic standard solutions of individual phenolics (starting from pure compounds, purity > 97% and provided from Extrasynthese, Lyon, France) were then injected. Sesamin (lignans), ferulic acid (hydroxycinnamic acids and other phenolic acids), luteolin (flavones and other remaining flavonoids), and tyrosol (hydroxytyrosols and tyrosol-equivalents) were used as representative of their respective classes. With this purpose, a linear fitting (not weighed and not forced to origin) was built and used for quantitative purposes. The abundance for each class was expressed as mg/kg equivalents of the reference compound within the same class.

### 2.9. Statistical Analysis

All analytical determinations were performed in triplicate. The reported results were expressed as mean ± standard deviation. Microsoft Excel 2013 (Microsoft Corporation, Redmond, WA, USA) was used for data analysis.

The Mass Profiler Professional B.12.06 (Agilent Technologies) was used for the elaboration of the untargeted UHPLC/QTOF data by unsupervised hierarchical cluster analysis (HCA), based on the fold-change heat map, as previously reported [17]. Thereafter, the raw metabolomic dataset was exported and elaborated into SIMCA 13 software (Umetrics, Malmo, Sweden) by supervised orthogonal projections to latent structures discriminant analysis (OPLS-DA) multivariate statistics. For the OPLS model, raw data were Log2 transformed to improve normal distribution, UV scaled to account for the wide range of intensities, and then analyzed. The variation between the groups was separated into predictive and orthogonal (i.e., related to technical and biological variation) components. The presence of outliers into the OPLS model was checked according to Hotelling’s T2 (i.e., the distance from the origin in the model), using 95% and 99% confidence limits for suspect and strong outliers, respectively. The model cross-validation was then carried out using CV-ANOVA (*p* < 0.01), whereas permutation testing (*n* = 100) was done to exclude overfitting. Model parameters, i.e., R^2^Y (goodness-of-fit) and Q^2^Y (goodness-of-prediction) were also recorded. Afterward, the variable selection method, namely, VIP (i.e., variable importance in projection) was used to evaluate the phenolic compounds most affected by the long-storage period, and to select those with the highest discrimination potential (VIP score > 1). Finally, a Fold-Change (FC) analysis was carried out considering the UHPLC/QTOF data from the UHPLC/QTOF data as compounds abundance ratio versus the control, in order to evaluate in detail, the trend and impact of the long-storage period on the main phenolic subclasses (i.e., tyrosols, phenolic acids, flavones, and lignans).

## 3. Results and Discussion

### 3.1. Acidity, Peroxide Values, and UV Spectrophotometric Indexes during Storage

In this study, the carotenoid-rich extract of *L. barbarum* was added to EVOO and stored for 28 weeks to evaluate the protective effect on some quality parameters during long-term storage.

**Figure 1:**
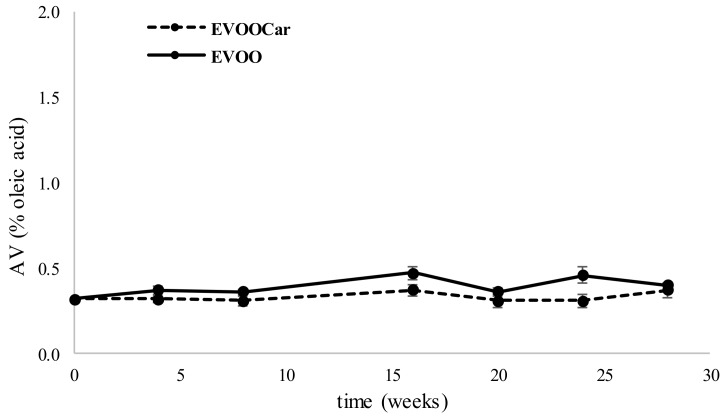
Trend of AV (% oleic acid, mean values) of extra virgin olive oil (EVOO) and carotenoid-enriched oil (EVOOCar) during long-term storage. Error bars refer to the standard deviations (*n* = 3).

Lipid deterioration in oils/fats may proceed essentially by enzyme-catalyzed hydrolytic cleavage, chemical hydrolytic cleavage, and atmospheric oxygen-driven oxidative lipid (autoxidation). Hydrolysis process results in the formation of free fatty acids (FFA) and partial esters of glycerol from triacylglycerols (mono- and diacylglycerols). Hydrolysis products decrease the stability of oils/fats and can be used to evaluate oil storage shelf-life, for example, measuring the AV. These types of alterations can be due to the presence of enzymes and water, as commonly happens with cloudy oils that are freshly obtained in oil mills. Figure 1 shows the trend of AV of EVOO and EVOOCAR, during long-term storage. Generally, the acidity of both oils remained within the maximum legal value acidity limit (0.8% for EVOO) established by European Regulation [33]. At the end of the storage, AV both for EVOO and EVOOCar were statistically significant different (*p* < 0.01) in respect to time zero, but no significant differences (*p* > 0.01) were observed between the two different oils.

**Figure 2:**
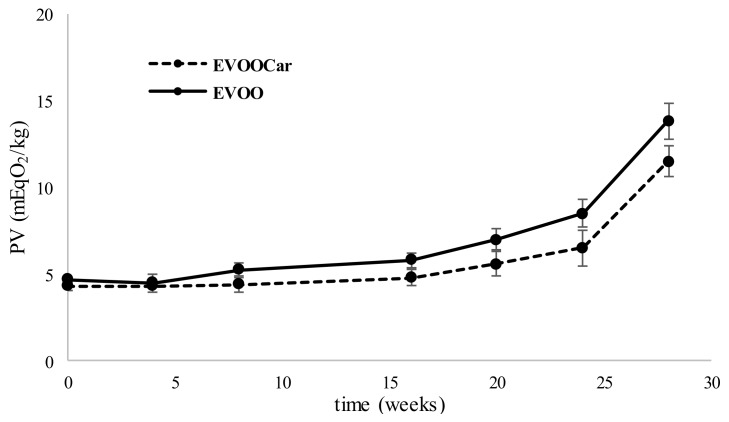
Trend of peroxide value (PV) (meq O_2_/kg oil, mean values) of EVOO and EVOOCar during long-term storage. Error bars refer to the standard deviations (*n* = 3).

During storage, food lipids can undergo oxidative phenomena, affecting the nutritional value of foods. Particularly deleterious for EVOO quality is the autoxidation process, enhanced by oxygen, light, heat, and heavy metals. Peroxides are intermediates of this type of reaction, so the PV determination is the official method, approved to evaluate the entity of oil primary oxidation, according to the European regulation [33]. Figure 2 shows the trend of PV of EVOO and EVOOCar during long-term storage. The PV of EVOO and EVOOCar remained fairly constant up to the 16th week, after which both oils showed an increasing trend of PV until the end of the storage (14 and 12 meqO_2_/kg for EVOO and EVOOCar, respectively). In any case, the PV of both oils remained within the maximum legal value acidity limit (20 meqO_2_/kg for EVOO) established by European Regulation [33]. At the end of the storage, the PV both for EVOO and EVOOCar were significantly different (*p* < 0.01) in respect to time zero. Furthermore, significant differences (*p* < 0.01) were also observed between the PV of the two different oils after the 28th week, in fact, the PV of EVOOCar was lower than the PV of EVOO. These results indicate that the carotenoid-rich extract is able to provide antioxidant protection against primary lipid oxidation. These results are in agreement with other literature data. For example, some researchers showed that during the first 13 weeks no remarkable variations were observed between oils added with lycopene and non-added oil. The authors also reported that at the end of the storage the PV was lower the maximum allowed by law for enriched oils, while it was around 40 meqO_2_/kg for non-added oil [26].

**Figure 3:**
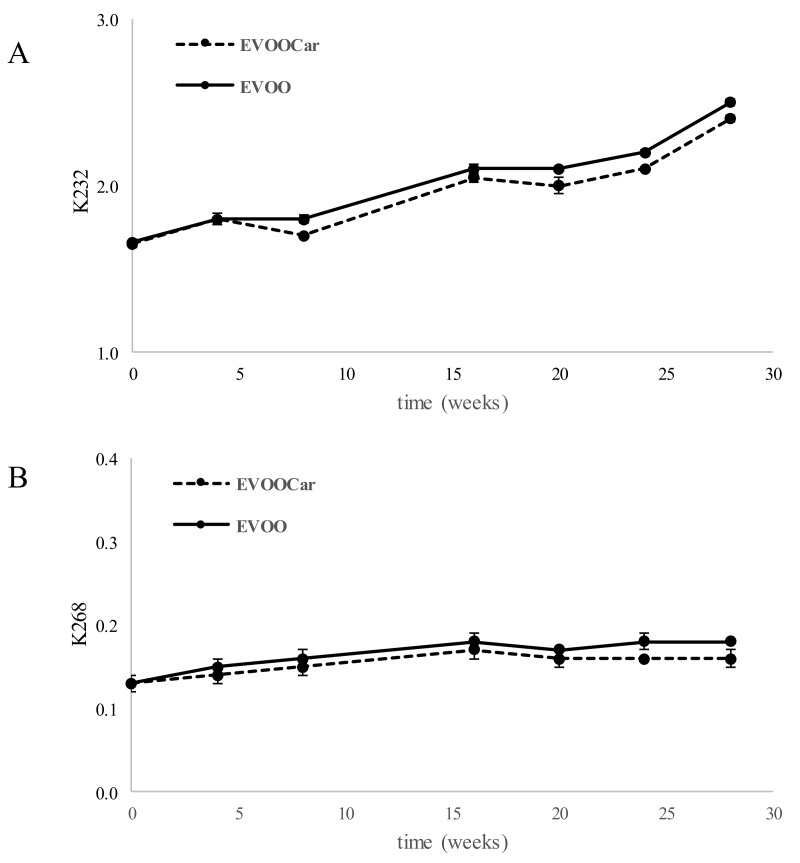
Trend of K232 (**A**) and K268 (**B**) of EVOO and EVOOCar during long-term storage (mean values). Error bars refer to the standard deviations (*n* = 3).

The determination of specific extinction values (K232 and K268) is the official method, established by European Regulation [33], for the evaluation of the degree of degradation of oils, linked to the production of peroxy radicals, unsaturated hydroperoxides, carbonyl conjugated compounds, and conjugated double bonds. Figure 3 shows the trends of K232 (A) and K268 (B) of EVOO and EVOOCar during long-term storage. During the storage, an increasing trend of K232 up to 2.50 and 2.40 was observed for the EVOO and EVOOCar, respectively, while K268 remained almost constant. The increasing trend showed for K232 was similar to the increasing trend of PV values at the same time, in fact, a good correlation between PV and K232 values (0.8532 and 0.8577, respectively for EVOO and EVOOCar) was found.

Other researchers also found that the behavior observed for K232 index was very similar to that found for PV, in EVOO stored for 21 months, showing a good correlation between the two parameters [34].

This aspect could be linked to the fact that K232 is due to the absorbance of primary oxidation products (i.e., peroxy radicals, hydroperoxides, conjugated double bonds), and that PV is the index of primary lipid oxidation. The values of K of both oils (EVOO and EVOOCar) remain within the maximum limit set by the European regulation 2016/2095 (2.50 for K232 and 0.22 for K268). At the end of the storage of EVOO and EVOOCar, significant differences (*p* < 0.01) were observed for K232 value with respect to time zero, but not for K268 value.

### 3.2. Acidic Composition during Storage

In EVOO and EVOOCar samples, the content of saturated fatty acids (SFA), mainly represented from palmitic, stearic and arachidic acids, was 14.6%. Both oils showed a high percentage of MUFA (78.6%), essentially represented by oleic acid, typical of EVOO. A lower percentage of polyunsaturated fatty acids (PUFA, 6.8%), represented by the essential FA linoleic and α-linolenic acids, was found. In details, the FA% compositions of EVOO and EVOOCar at different storage times are shown in the Supplementary Materials (Table S1). The EVOO acidic composition is in agreement with other literature data [16,17,35]. The results of this investigation show that EVOO acidic compositions did not change after 28 weeks (*p* > 0.05), therefore, it is possible to conclude that during long-term storage the natural antioxidants of EVOO are able to protect unsaturated FA from oxidative modifications. As expected, the fatty acid composition was also unchanged (*p* > 0.05) in EVOOCar, for the presence of antioxidants naturally present in EVOO and of carotenoid-rich extract. On the contrary, in a previous paper it has been reported that, during EVOO frying, MUFA (oleic acid) and PUFA (linoleic and α-linolenic acids) showed a significant percentage loss, while they remained constant or slightly changed in EVOOCar [17]. Other researchers reported that the addition of sesame seed oil caused a significant change in the fatty acid composition in EVOO, as a function of mixture levels and storage period. Other literature data, regarding the effects of long-term storage on vegetable oils, are in agreement with our results [16]. For example, the fatty acid content of flaxseed oil cake, enriched in phenols, did not change during storage for up 6 months, maintaining an optimal *n*-6/*n*-3 ratio [36]. Few changes were also observed in the unsaturated fatty acid composition of virgin olive oils during the 21-month storage period [34].

The results obtained show that EVOO, during the storage for 28 weeks, maintains its nutritional characteristics and health properties, due to the high content of oleic acid and a good *n*-6/*n*-3 ratio [37].

### 3.3. Content of α-Tocopherol and Carotenoids during Storage

Another objective of this study was to investigate the changes in the carotenoidic fraction and α-tocopherol of EVOO during long-term storage and to evaluate the protective effect of Goji extract added to EVOO.

α-Tocopherol, the main compound of tocopherols, represents the major antioxidant found in olive oil, in fact, its natural concentration can reach up to 300 ppm [38]. Since vegetable fats and oils are the main sources of vitamin E-active compounds in the human diet, it is important to preserve this molecule against degradation during storage.

**Figure 4:**
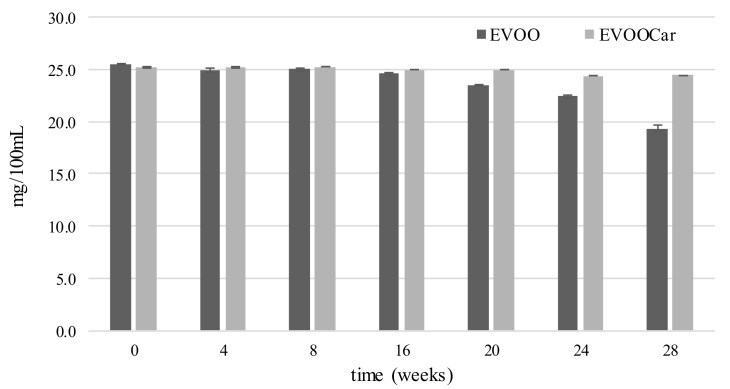
Trend of α-tocopherol content (mg/100 mL, mean values) of EVOO and EVOOCar during long-term storage. Error bars refer to the standard deviations (*n* = 3).

Figure 4 shows the trend of α-tocopherol content in EVOO and in EVOOCar during the storage. The initial α-tocopherol levels were fairly constant for both oils up to the 12th week. After this time, the α-tocopherol content of EVOO decreased significantly (*p* < 0.01) until the end of storage, differently from EVOOCar, that showed similar content (*p* > 0.05) between zero time and the 28th week. At the end of the storage, a higher content (*p* < 0.01) of α-tocopherol in EVOOCar in respect to EVOO (24.4 vs. 19.4 mg/100 mL) was observed, highlighting the protective effect of Goji carotenoids on α-tocopherol levels. Different results have been obtained monitoring α-tocopherol content in EVOO and EVOO during frying [17]. In fact, a significant decrease of α-tocopherol was observed both in EVOO and EVOOCar, already after 20 min of frying. Other researchers also reported that tocopherol content markedly decreased in sunflower oil during accelerated storage and coriander essential oil addition significantly limited this loss [8]. On the contrary, the results of this research are in agreement with those reported by Montesano and collaborators, which observed that the addition of lycopene to EVOO, in suitable concentrations, preserved the major part of α-tocopherol initially present in the oil [26].

Since α-tocopherol possess important vitamin and antioxidant activity, the fact that the addition of Goji carotenoids maintains its content is highly relevant, since both important biological properties are preserved in the olive oils.

Carotenoids are naturally occurring organic pigments, widely present in fruits, vegetables, plants, and algae, characterized by important healthy properties [39,40,41,42]. Some authors have reported the addition of carotenoids to different kinds of edible oils. For example, the stability of EVOO added with carotenoid-rich extracts from *Scenedesmus almeriensis* at different concentrations (0.1 and 0.21 mg/mL) has been studied. The authors reported that the quality of olive oils was improved, since β-carotene and lutein contents increased considerably [43]. Some researchers obtained a virgin olive oil enriched with lutein and zeaxanthin from *Spinacia oleracea* as a potential functional food [44]. Carotenoids (β-carotene and astaxanthin) have been added to refined olive oils, and it has been reported that only astaxanthin showed a protective effect on olive oil for up to 10 h of thermal treatment [5].

In this research, a low concentration of Goji carotenoids has been added to EVOO to avoid the pro-oxidant effect [5], according to a previous work [6]. Table 1 shows the content (mg/100 mL) of lutein, β-carotene, and zeaxanthin dipalmitate, during the storage of EVOO and EVOOCar. The main pigment of EVOO is the lutein (3,3′-dihydroxy-α-carotene), followed by β-carotene. The content of lutein observed in the starting EVOO was around 1.0 mg/100 mL, a considerably high value in respect to the values reported in the literature for other EVOO samples [44]. At the end of the storage, the content of β-carotene and lutein in EVOO and EVOOCar showed slight decreases in respect to time zero. Zeaxanthin dipalmitate, the most abundant carotenoid compound of Goji berries [24], decreased slightly in EVOOCar until the end of the storage, showing statistical differences in respect to time zero (*p* < 0.05). A more drastic decrease of zeaxanthin dipalmitate content was observed in EVOOCar subjected to frying process, already after 10 min of thermal treatment [17].

It is interesting to highlight that zeaxanthin dipalmitate shows enhanced bioavailability compared to free zeaxanthin [45], for which EVOO enrichment with *L. barbarum* extract might represent a valuable strategy to improve oil stability while providing health-promoting compounds.

### 3.4. UHPLC/QTOF Screening of Phenolic Compounds during Storage

The changes of the polyphenol profiles during long-term storage of EVOO and EVOOCar were investigated by means of a UHPLC/QTOF mass spectrometry approach. In particular, this high-resolution method annotated (i.e., according to a Level 2 of accuracy, as set out by the COSMOS Metabolomics Standard Initiative) more than 400 compounds, consisting of 71 anthocyanins, 95 flavones, 93 flavonols, 29 lignans, 14 alkylphenols, 65 tyrosols, 100 phenolic acids, and 10 stilbenes. The polyphenols annotated with this approach are reported in the Supplementary Materials (Table S2), together with composite mass spectra (mass and abundance combination) and annotation scores. However, considering the typical EVOO phenolic composition, as set out in both Phenol-Explorer 3.6 (i.e., the most comprehensive database on polyphenol content in foods) and the literature [17], we highlighted the changes of the most important phenolic classes, being flavones, phenolic acids (i.e., hydroxybenzoics, hydroxycinnamics, and hydroxyphenylacetics), lignans, and tyrosol equivalents.

In this regard, the phenolic acids class was characterized by 25 hydroxybenzoics, 68 hydroxycinnamics, and 5 hydroxyphenylacetics. Among hydroxybenzoics, isomeric forms of dihydroxybenzoic acid were very frequent, followed by syringic and vanillic acids. Besides, looking to the hydroxycinnamics subclass, an abundance of isomeric forms of coumaric and ferulic acids was noticed. According to the phenolic composition reported for EVOO, 4-hydroxyphenylacetic acid and homovanillic acid were the most represented among the hydroxyphenylacetic acids (Table S2). Turning to tyrosols, the 3,4-DHPEA-EA and *p*-HPEA-EDA were particularly abundant in the EVOO samples, followed by oleuropein, ligstrosides and their aglycones, and tyrosols and hydroxytyrosols. Interestingly, the distribution of these compounds was in compliance with the EVOO composition reported in Phenol-Explorer. Moreover, with respect to the class of flavonoids, the flavones apigenin and luteolin (together with their glycosidic forms) were the most frequent compounds annotated. Finally, in our experimental conditions, two of the most reported lignans in EVOO were also detected, being pinoresinol and 1-acetoxypinoresinol (Table S2).

**Figure 5:**
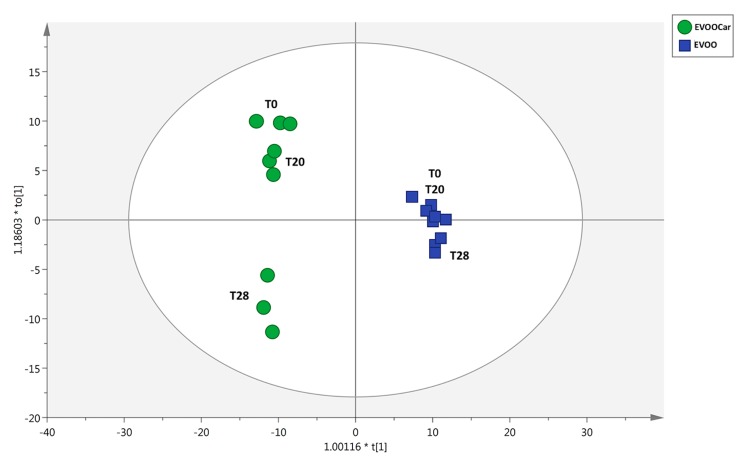
Orthogonal projection to latent structures discriminant analysis (OPLS-DA) on both EVOO and EVOOCar during long-term storage, considering time 0, 20, and 28 weeks. Individual replications are given in the class prediction model score plot.

Afterward, the multivariate supervised orthogonal projection to latent structures discriminant analysis (OPLS-DA) was carried out in order to investigate the chemical changes induced by the long-term storage. In particular, the OPLS-DA model was built considering three time points, being 0, 20, and 28 weeks of storage. The OPLS-DA score plot obtained by using this multivariate approach is reported in Figure 5. This OPLS model was characterized by excellent model parameters, being R^2^Y = 0.99 (goodness of fit) and Q^2^Y = 0.88 (goodness of prediction). Besides, the model was cross-validated and inspected for outliers, as reported in Table S2. Looking at the output obtained, great discrimination among the two group of samples (i.e., EVOO and EVOOCar) could be observed, likely due to the addition of Goji carotenoid extract. In this regard, the major differences could be outlined in EVOOCar after 28 weeks of storage (Figure 5). Therefore, a second model was built to exclusively compare EVOO and EVOOCar at 28 weeks of storage (Table S2) in order to confirm the changes promoted by the addition of carotenoid extract at this time-point. The VIP selection method was used to evaluate the importance of the variables in the projection of the OPLS-DA model, and then to outline the phenolic classes most affected by the addition of the Goji carotenoid. Interestingly, the VIP approach identified 70 compounds able to discriminate the two different groups of samples, belonging to the subclasses of phenolic acids, flavones, and tyrosol equivalents. These VIP markers are reported in Table 2 together with their individual score (>1), log fold-change (FC) values, and the corresponding up/down-regulation. As can be observed from Table 2, the majority of flavones equivalents were characterized by an overall up-regulation (75%) when considering the comparison EVOOCar vs. EVOO during 28 weeks of storage. In particular, 10 compounds were characterized by logFC values higher than 15, such as naringin 6’-malonate and neodiosmin. When considering the other VIP markers for the same comparison (i.e., EVOOCar vs. EVOO), tyrosol equivalents (i.e., 81%) showed the same up-regulation trend. In fact, only a few compounds were down-regulated, with 10 compounds possessing logFC values higher than 10 (such as *p*-HPEA-AC).

**Figure 6:**
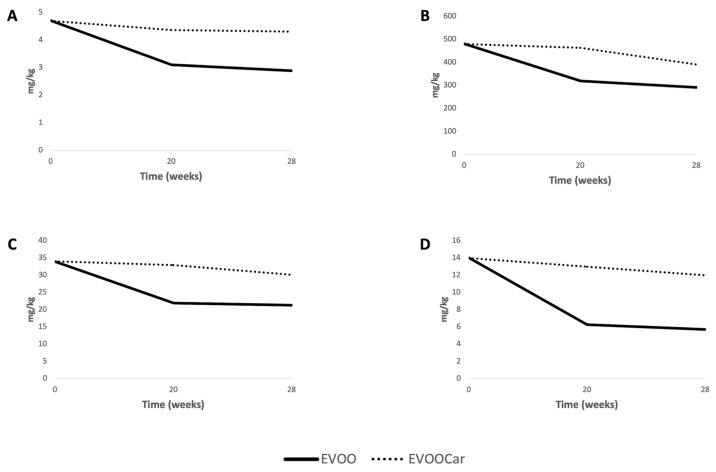
Semi-quantitative values and trends during long-term storage (0, 20 and 28 weeks) for the main phenolic classes of EVOO and EVOOCar. Data are provided from UHPLC-ESI/QTOF-MS and reported as mg/kg equivalents. **A** = phenolic acids; **B** = tyrosol equivalents; **C** = flavones; **D** = lignans.

The semi-quantitative analysis of the major phenolic classes (Figure 6) clearly depicted the impact of Goji carotenoids on the entire phenolic profile during long-term storage. In fact, the phenolic acids content in EVOOCar remained between 4.70 and 4.35 mg/kg, almost stable during the entire time of storage. Instead, EVOO showed deep changes during storage, recording a 39% reduction after 28 weeks. Similarly, the tyrosol equivalents content of EVOOCar moved from 480.0 to 463.0 after 20 weeks, then presented a slight decrease at 28 weeks (−19%). In contrast, EVOO sample was characterized by a general decrease in tyrosol equivalents content, reaching 290.00 mg/kg after 28 weeks (−40%). When considering the other phenolic classes investigated by exploiting the metabolomics-based approach, the flavone content showed clear semi-quantitative differences between EVOOCar and EVOO, particularly marked after 20 weeks, at 35 and 20 mg/kg, respectively. Finally, lignans were found to be the most affected class of compounds during storage. In fact, after 20 weeks, the abundance of these compounds drastically decreased in EVOO sample when compared to EVOOCar, at −55% vs. −7% reduction, respectively.

In this work, we demonstrated that the addition of *L. barbarum* carotenoids to EVOO during long-term storage could preserve from oxidation processes its main polyphenol composition (i.e., flavones, lignans, phenolic acids, and tyrosol equivalents). In recent years, there has been increasing interest in the prediction/evolution of antioxidant compounds characterizing EVOO during storage [46]. In particular, research efforts aimed to evaluate changes of antioxidant compounds during storage at room and in darkness conditions [46]. Overall, total phenolic content in oil varies from 50 to 1000 mg/kg, depending on cultivar, origin, agronomic techniques, storage condition, and other factors [17,31,47]. However, it is important to emphasize that results from different studies on EVOO polyphenols are difficult to compare because of the variety of methods proposed for their determination. For example, most works used spectrophotometric methods, such as Folin-Ciocalteu, to determine changes in polyphenols during EVOO and VOO storages [46]; however, these methods are not only specific for phenols, thus hindering the comparability between similar studies. That is why LC-MS are now recommended to quantify antioxidant compounds in food matrices [48].

Some researchers investigated the evolution in the phenolic composition of oils during 21 months of storage at room temperature, finding that the reduction of total phenolics ranged from 43% up to 73% and was higher in samples whose initial phenolic contents were greater [34]. In particular, storage can affect the phenolic profile of oil through oxidative stress phenomena and the consequent formation of oxidized phenolics [49]. For example, 3,4-DHPEA and *p*-HPEA-EA are reported to increase during long-term storage, and therefore the ratio fresh/oxidized phenolics has been proposed as a marker of the freshness/aging of different oils, while hydroxytyrosol concentration is reported to decrease and closely correlated with the oxidative stability of the olive oil [46]. Accordingly, we found that 3,4-DHPEA-EA and *p*-HPEA-EA increased two-fold in EVOOCar after 28 weeks of storage, while hydroxytyrosol showed a decreasing trend of −25% (Table S2).

In a previous work [17], we evaluated the changes of the main polyphenol classes in EVOO added with Goji carotenoids during frying; in particular, we demonstrated that the decrease of phenolics was lower in EVOOCar than in EVOO during thermal processing, thus postulating the exploitation of a carotenoid extract from *L. barbarum* to improve the oxidative stability of EVOO. In recent years, different sources of natural bioactive ingredients and extraction conditions have been described for the development of novel functional virgin olive oil [50]. In this regard, the most exploited sources derived from the same olive tree (mainly leaves or olive pomace) and from the use of plants and vegetables, mainly herbs and spices. Therefore, to the best of our knowledge, this is the first work highlighting the effect of *L. barbarum* carotenoids as a valuable tool to preserve the phenolic profile of EVOO during long-term storage. In a previous work, it has been demonstrated that carotenoid-rich extracts from microalgae added to VOO can help in improving the oxidative stability and, as a consequence, olive oil shelf-life and nutritional value [43]. However, the same authors concluded that the assessment of the stability of these enriched-oils represents a challenge, thus requiring further studies. In particular, scientific research must also focus on determining the exact amount of protective extracts that should be incorporated to not alter oil quality parameters and to improve its health-promoting properties [50].

## 4. Conclusions

The results of this work suggest that the addition of a Goji carotenoid-rich extract to EVOO could be useful to produce novel nutraceutical oil, with improved oxidative stability, shelf-life, and nutritional value in respect to the original one. The addition of natural antioxidants, for example, Goji carotenoids with well-known healthy properties, opens the way to the market of enriched oils with a nutritional-added value. To this aim, in a successive phase of the research, a wider EVOO sampling should be considered, taking into account the variability of EVOO composition. The UHPLC/QTOF-MS-based metabolomics analysis of EVOO and EVOOCar was highly informative, allowing the discrimination between the two series of oils (added and not added), during long-term storage. The results of this work suggest that UHPLC/QTOF-MS-based metabolomics analysis is a suitable approach to detect biomarkers, including phenolic compounds, useful in the field of food quality/safety, that indicate degradation resulting from the long-term storage of EVOO.

## Figures and Tables

**Table 1:** Content (mg/100 mL, mean values ± standard deviation, *n* = 3) of lutein, β-carotene, and zeaxanthin dipalmitate in EVOO and EVOOCar during storage.

Time (Weeks)	Lutein		β-carotene		Zeaxanthin Dipalmitate
	EVOO	EVOOCar	EVOO	EVOOCar	EVOOCar
0	0.99 ± 0.01	0.99 ± 0.03	1.03 ± 0.02	1.03 ± 0.04	1.59 ± 0.08
4	0.95 ± 0.03	0.98 ± 0.04	0.95 ± 0.04	1.01 ± 0.05	1.50 ± 0.09
8	0.95 ± 0.02	0.97 ± 0.02	0.94 ± 0.01	1.00 ± 0.02	1.48 ± 0.01
12	0.94 ± 0.02	0.96 ± 0.03	0.92 ± 0.02	1.10 ± 0.03	1.47 ± 0.04
16	0.92 ± 0.02	0.96 ± 0.04	0.94 ± 0.03	1.00 ± 0.04	1.47 ± 0.03
20	0.90 ± 0.01	0.95 ± 0.02	0.87 ± 0.01	0.98 ± 0.03	1.28 ± 0.01
24	0.88 ± 0.02	0.94 ± 0.01	0.85 ± 0.01	0.95 ± 0.02	1.31 ± 0.03
28	0.84 ± 0.02	0.90 ± 0.03	0.84 ± 0.03	0.93 ± 0.02	1.30 ± 0.02

**Table 2:** Phenolic compounds discriminating EVOOCar vs. EVOO samples after 28 weeks of the storage process, grouped into phenolic chemical subclasses, as resulted by variable importance in projections (VIP) of OPLS-DA, together with the corresponding logFC regulation.

Phenolic Class	Phenolic Subclass	Compound	VIP Score	LogFC	Regulation
Flavonoids	Flavanones	Naringin 6’-malonate	1.34	19.01	up
		Poncirin	1.20	18.59	up
		Didymin	1.20	18.59	up
		Naringenin	1.11	2.46	up
		Eriocitrin	1.11	0.64	up
		Neoeriocitrin	1.11	0.64	up
		Naringenin 7-*O*-glucoside	1.08	0.26	up
		Naringin 4’-*O*-glucoside	1.08	0.26	up
		Eriodictyol 7-*O*-glucoside	1.04	−5.85	down
		Pinocembrin	1.03	−0.48	down
		6-Geranylnaringenin	1.03	2.07	up
	Flavones	Cirsilineol	1.34	0.12	down
		Eupatorin	1.34	0.12	down
		Pebrellin	1.34	0.12	down
		Luteolin 7-*O*-malonyl-glucoside	1.34	0.14	up
		Apigenin 7-*O*-diglucuronide	1.34	0.16	up
		Apigenin	1.34	0.13	up
		Luteolin 7-*O*-(2-apiosyl-glucoside)	1.33	0.21	up
		7,3’,4’-Trihydroxyflavone	1.33	0.24	up
		Baicalein	1.33	0.24	up
		Apigenin 7-*O*-(6’’-malonyl-apiosyl-glucoside)	1.32	0.30	down
		Gardenin B	1.29	0.55	up
		Chrysoeriol 7-*O*-apiosyl-glucoside	1.28	0.59	up
		Luteolin 7-*O*-rutinoside	1.28	0.59	up
		Apigenin 6,8-di-*C*-glucoside	1.28	0.59	up
		Chrysoeriol 7-*O*-(6’’-malonyl-glucoside)	1.27	0.52	up
		Neodiosmin	1.24	0.64	up
		Diosmin	1.24	0.64	up
		Nepetin	1.24	0.61	down
		Tetramethylscutellarein	1.18	0.94	up
		Rhoifolin 4’-*O*-glucoside	1.17	0.92	up
		Apigenin 6-*C*-glucoside	1.03	0.99	down
Lignans	Lignans	1-Acetoxypinoresinol	1.02	9.09	up
Other polyphenols	Hydroxybenzaldehydes	*p*-Anisaldehyde	1.26	1.34	up
		Syringaldehyde	1.07	16.29	up
	Hydroxycoumarins	Umbelliferone	1.30	13.69	up
		4-Hydroxycoumarin	1.30	13.69	up
		Scopoletin	1.20	0.31	up
		Mellein	1.05	0.81	up
	Hydroxyphenylpropenes	Acetyl eugenol	1.06	1.22	up
	Naphtoquinones	Juglone	1.21	0.37	up
	Phenolic terpenes	Thymol	1.33	18.01	up
		Rosmadial	1.24	8.68	up
Tyrosols	Tyrosols	Demethyloleuropein	1.33	−2.68	down
		*p*-HPEA-AC	1.28	13.59	up
		Oleuropein	1.25	−3.40	down
		*p*-HPEA-EDA	1.20	−1.57	down
		Tyrosol	1.04	0.85	up
Phenolic acids	Hydroxybenzoic acids	Ellagic acid	1.34	16.26	up
		Ellagic acid arabinoside	1.33	16.85	up
		Syringic acid	1.28	−0.41	down
		Gallic acid ethyl ester	1.28	−0.41	down
		Ellagic acid acetyl-xyloside	1.27	16.89	up
		Ellagic acid acetyl-arabinoside	1.27	16.89	up
		Gallic acid 3-*O*-gallate	1.21	0.47	up
		Galloyl glucose	1.09	−16.45	down
		Valoneic acid dilactone	1.08	11.92	up
	Hydroxycinnamic acids	*p*-Coumaroyl malic acid	1.34	0.14	up
		Caffeoyl aspartic acid	1.33	0.27	down
		Caffeic acid	1.30	0.36	down
		3,5-Diferuloylquinic acid	1.29	0.45	up
		1,2-Diferuloylgentiobiose	1.28	0.51	up
		24-Methylcholestanol ferulate	1.28	0.48	down
		Cinnamoyl glucose	1.24	0.56	up
		1-Sinapoyl-2-feruloylgentiobiose	1.20	0.72	up
		Chicoric acid	1.14	0.85	up
		8-*O*-4’-Dehydrodiferulic acid	1.06	1.14	up
		5-8’-Dehydrodiferulic acid	1.06	1.14	up
	Hydroxyphenylacetic acids	Homovanillic acid	1.08	16.29	up

## Supplementary Materials

The following are available online at https://www.mdpi.com/2304-8158/8/6/179/s1, Table S1: The FA% compositions of EVOO and EVOOCar at different storage times, Table S2: Polyphenol compounds obtained by metabolomics approach, together with composite mass spectra (mass and abundance combination) and annotation scores.
