# Supplementary material for: Untargeted Metabolomics to Evaluate the Stability of Extra-Virgin Olive Oil with Added Lycium barbarum Carotenoids during Storage
Source: Foods. 2019 May 28;8(6):179. doi: 10.3390/foods8060179 (PMC6616970; doi:10.3390/foods8060179)
Supplement: Supplementary file 1 [file foods-08-00179-s001.zip › Supplementary S1.docx]

**Table S1.** The FA % compositions of EVOO and EVOOCar at different storage times.

|  | **Time (Weeks)** | | | | | | |
| --- | --- | --- | --- | --- | --- | --- | --- |
| **FA** | **0** | **4** | **8** | **16** | **20** | **24** | **28** |
| **EVOO** | | | | | | | |
| C16:0 | 11.9 ± 0.0 | 12.2 ± 0.1 | 12.2 ± 0.1 | 12.8 ± 0.2 | 12.1 ± 0.0 | 12.7 ± 0.0 | 12.4 ± 0.1 |
| C16:1 (*n*-9 + *n*-7) | 0.4 ± 0.0 | 0.7 ± 0.0 | 0.6 ± 0.0 | 0.6 ± 0.0 | 0.8 ± 0.0 | 0.7 ± 0.0 | 0.6 ± 0.0 |
| C18:0 | 2.1 ± 0.1 | 2.1 ± 0.0 | 2.2 ± 0.0 | 2.1 ± 0.1 | 2.1 ± 0.1 | 2.1 ± 0.1 | 2.1 ± 0.0 |
| C18:1 (*n*-9 + *n*-7) | 78.1 ± 0.1 | 76.9 ± 0.3 | 76.7 ± 0.2 | 76.4 ± 0.3 | 77.0 ± 0.3 | 76.6 ± 0.3 | 77.1 ± 0.2 |
| C18:2 (*n*-6) | 6.1 ± 0.0 | 6.6 ± 0.0 | 6.7 ± 0.2 | 6.5 ± 0.1 | 6.5 ± 0.1 | 6.5 ± 0.1 | 6.5 ± 0.1 |
| C20:0 | 0.4 ± 0.0 | 0.4 ± 0.0 | 0.3 ± 0.0 | 0.4 ± 0.0 | 0.4 ± 0.0 | 0.4 ± 0.0 | 0.8 ± 0.0 |
| C18:3 (*n*-3) | 0.8 ± 0.0 | 0.8 ± 0.0 | 0.8 ± 0.0 | 0.8 ± 0.0 | 0.8 ± 0.0 | 0.8 ± 0.0 | 0.4 ± 0.0 |
| C20:1 | 0.4 ± 0.0 | 0.4 ± 0.0 | 0.5 ± 0.0 | 0.4 ± 0.0 | 0.4 ± 0.0 | 0.4 ± 0.0 | 0.1 ± 0.0 |
| **EVOOCar** | | | | | | | |
| C16:0 | 11.9 ± 0.2 | 12.2 ± 0.2 | 11.6 ± 0.1 | 12.3 ± 0.1 | 12.4 ± 0.2 | 12.8 ± 0.4 | 12.1 ± 0.2 |
| C16:1 (*n*-9 + *n*-7) | 0.4 ± 0.0 | 0.5 ± 0.0 | 0.4 ± 0.0 | 0.4 ± 0.0 | 0.4 ± 0.0 | 0.5 ± 0.0 | 0.5 ± 0.0 |
| C18:0 | 2.1 ± 0.1 | 2.1 ± 0.0 | 2.1 ± 0.0 | 2.1 ± 0.1 | 2.0 ± 0.1 | 2.1 ± 0.0 | 2.0 ± 0.2 |
| C18:1 (*n*-9 + *n*-7) | 78.1 ± 0.3 | 77.8 ± 0.3 | 78.3 ± 0.2 | 77.8 ± 0.4 | 77.7 ± 0.3 | 77.3 ± 0.3 | 78.0 ± 0.4 |
| C18:2 (*n*-6) | 6.0 ± 0.2 | 5.9 ± 0.1 | 6.0 ± 0.0 | 5.9 ± 0.1 | 5.9 ± 0.3 | 5.8 ± 0.1 | 5.9 ± 0.2 |
| C20:0 | 0.4 ± 0.0 | 0.4 ± 0.0 | 0.4 ± 0.0 | 0.4 ± 0.0 | 0.3 ± 0.0 | 0.7 ± 0.0 | 0.3 ± 0.0 |
| C18:3 (*n*-3) | 0.8 ± 0.0 | 0.8 ± 0.0 | 0.8 ± 0.0 | 0.8 ± 0.0 | 0.7 ± 0.0 | 0.3 ± 0.0 | 0.8 ± 0.0 |
| C20:1 | 0.5 ± 0.0 | 0.4 ± 0.0 | 0.4 ± 0.0 | 0.4 ± 0.0 | 0.4 ± 0.0 | 0.4 ± 0.0 | 0.4 ± 0.0 |
